# Do Personality Traits Predict Individual Differences in Excitatory and Inhibitory Learning?

**DOI:** 10.3389/fpsyg.2013.00245

**Published:** 2013-05-08

**Authors:** Zhimin He, Helen J. Cassaday, Charlotte Bonardi, Peter A. Bibby

**Affiliations:** ^1^School of Psychology, University of NottinghamNottingham, UK

**Keywords:** conditioned inhibition, behavioral activation, behavioral inhibition, neuroticism

## Abstract

Conditioned inhibition (CI) is demonstrated in classical conditioning when a stimulus is used to signal the omission of an otherwise expected outcome. This basic learning ability is involved in a wide range of normal behavior – and thus its disruption could produce a correspondingly wide range of behavioral deficits. The present study employed a computer-based task to measure conditioned excitation and inhibition in the same discrimination procedure. CI by summation test was clearly demonstrated. Additionally summary measures of excitatory and inhibitory learning (difference scores) were calculated in order to explore how performance related to individual differences in a large sample of normal participants (*n* = 176 following exclusion of those not meeting the basic learning criterion). The individual difference measures selected derive from two biologically based personality theories, Gray’s (1982) reinforcement sensitivity theory and Eysenck and Eysenck (1991) psychoticism, extraversion, and neuroticism theory. Following the behavioral tasks, participants completed the behavioral inhibition system/behavioral activation system (BIS/BAS) scales and the Eysenck personality questionnaire revised short scale (EPQ-RS). Analyses of the relationship between scores on each of the scales and summary measures of excitatory and inhibitory learning suggested that those with higher BAS (specifically the drive sub-scale) and higher EPQ-RS neuroticism showed reduced levels of excitatory conditioning. Inhibitory conditioning was similarly attenuated in those with higher EPQ-RS neuroticism, as well as in those with higher BIS scores. Thus the findings are consistent with higher levels of neuroticism being accompanied by generally impaired associative learning, both inhibitory and excitatory. There was also evidence for some dissociation in the effects of behavioral activation and behavioral inhibition on excitatory and inhibitory learning respectively.

## Introduction

Conditioned inhibition (CI) is an associative learning phenomenon in which a stimulus (known as a conditioned inhibitor) is used to signal the omission of an otherwise expected outcome. For example, if a conditioned stimulus (CS) A signals a reinforcing unconditioned stimulus (US), and then after a number of training trials A is presented with another CS B, but now the expected US does not follow, participants learn that B indicates no US; in other words B is a conditioned inhibitor (Pavlov, 1927). Associative learning is a ubiquitous process of evolutionary advantage. It is not only fundamental, being found in all vertebrates, but has been argued to underlie many more sophisticated cognitive processes in both animals and humans. CI is therefore likely to be involved in a broad range of normal behavior – and thus its disruption could produce a wide range of behavioral deficits.

Lack of inhibitory control has been argued to lie at the heart of impulsivity (Buss and Plomin, 1975), which is a core feature of a number of psychological conditions, such as schizophrenia, and personality disorders (PDs), especially within forensic populations (Hare et al., 1991; Munro et al., 2007). Highly impulsive individuals have difficulty withholding responding, as demonstrated by poor performance in laboratory-based behavioral tasks such as Go/No-Go (Visser et al., 1996; Logan et al., 1997; Enticott et al., 2006). However, these established tasks measure participants’ ability to inhibit pre-potent motor responses, and are generally thought to involve the inhibition of stimulus-response associations. In contrast, relatively little research has explored the inhibition of stimulus–stimulus (CS-US) associations (formally CI) in populations likely to differ in impulsivity. To our knowledge, the only exception is evidence from our own work – we have reported individual variation in CI in relation to medication (Kantini et al., 2011a,b), level of dangerousness and severity of PDs (He et al., 2011), as well as in relation to symptom profile in schizophrenia (He et al., 2012).

However, such clinical samples are difficult to recruit in large numbers, and it is especially hard to isolate larger samples “uncontaminated” by confounded conditions – such as participants with Tourette syndrome in the absence of ADHD (Kantini et al., 2011a) or vice versa (Kantini et al., 2011b; see also He et al., 2011, 2012). Thus an alternative approach would be to examine the relationship between CI learning and individual differences in personality traits in the general population (Migo et al., 2006). This previous study used the behavioral inhibition system/behavioral activation system (BIS/BAS) scale (Gray, 1981; Carver and White, 1994), as well as a measure of schizotypy, and CI was measured using an earlier task variant without full behavioral controls (as here). Probably the most widely used model of normal personality is the “Big Five” (Costa and McCrae, 1992) which includes extraversion and neuroticism, but not psychoticism which we wished to examine given our findings in clinical groups (He et al., 2011, 2012). The present study set out to examine CI in a large sample of normal participants using questionnaires designed to tap personality traits relating to comparative analyses of brain function, specifically in terms of differences in conditionability. Accordingly, participants were administered the Eysenck personality questionnaire revised short scale (EPQ-RS; Eysenck et al., 1985), as well as the BIS/BAS (Gray, 1981; Carver and White, 1994).

Eysenck’s personality scales initially captured impulsivity in relation to extraversion and, in the revised version of the theory, as a core feature of its psychoticism dimension (Eysenck and Eysenck, 1991). Building on Eysenck’s theory, the BIS/BAS scales were devised as orthogonal measures of anxiety and impulsivity respectively (Gray, 1981; Carver and White, 1994; Pickering and Gray, 1999). More specifically, Gray (1970, 1972, 1982, 1990) argued that the BAS measures activity in a system sensitive to signals of reward, which may, in predisposed individuals, elicit impulsive or antisocial tendencies. Consistent with this analysis, impulsivity has been related to enhanced learning about signals for reward (Avila et al., 2008), and neuroimaging evidence suggests that BAS activation is associated with the processing of positive stimuli in reward-related areas (albeit with some inconsistencies which may relate to the relative salience of the images in use for different individuals; Beaver et al., 2006; Avila et al., 2008). In contrast, the BIS relates to activity in a system responding to signals for non-reward, punishment, and novelty, producing inhibition of movement toward goals and other symptoms of anxiety. According to Gray’s theory, BIS and BAS activity are independent, and dissociations in the relationship between anxiety and impulsivity and (for example) the processing of threat-relevant stimuli have in fact been demonstrated (Putman et al., 2004). Moreover, in anxiety disorders, aspects of impulsivity are negatively related to behavioral inhibition (Pierò, 2010; Snorrason et al., 2011); as would be expected, impulsivity has been suggested to result from deficient behavioral inhibition (Fowles, 1987). Thus there are both theoretical and empirical grounds to suggest that anxiety and impulsivity are inversely related.

Later refinement of the original behavioral inhibition theory (Gray and McNaughton, 2000) resulted in the introduction of sub-scales to the BIS (Carver and White, 1994), to capture the distinction between fear and anxiety (with BIS-anxiety and BIS-FFFS sub-scales; Gray and McNaughton, 2000; Smillie et al., 2006). Confirmatory factor analysis supports this revision to the theory and shows how the new model (with BIS-anxiety and BIS-FFFS sub-scales) relates to Eysenck’s theory; for example, neuroticism relates to BIS-anxiety as well as the BIS-FFFS sub-scale, whereas psychoticism relates to BIS-anxiety and BAS (Heym et al., 2008).

Thus, although they do not measure it directly, impulsivity is nonetheless captured by these general theories of personality. The broader predispositions measured by the EPQ-RS and the BIS/BAS also relate to disorder, in that EPQ-RS neuroticism and BIS scores specifically measure susceptibility to anxiety-related conditions (Eysenck, 1957, 1967; Eysenck and Eysenck, 1976a,b). More generally, disinhibition as a mechanism for impulsivity could potentially apply to a variety of behavioral disorders to which anxiety is less central, including antisocial behavior, and psychopathy (He et al., 2011). Although psychopathy is a clinical condition rather than a personality trait, it is nonetheless related to the personality trait of psychoticism (Eysenck and Eysenck, 1976b). In relation to underlying neuropsychological substrates, both have been argued to result from dysfunction in the BIS (Gray, 1972, 1982).

This relationship has been further specified in terms of the BIS-FFFS, which mediates avoidance or escape in response to fear (Gray and McNaughton, 2000; Smillie et al., 2006). Low and high BIS-FFFS activity have been suggested to characterize primary and secondary psychopathy respectively, while secondary psychopaths are said also to be characterized by high BAS activity (Corr, 2010). Relatedly, statistical analyses of scores from a normal population have recently confirmed that high psychoticism scores are associated with reduced fear and anxiety (also characteristic of primary psychopathy) and increased impulsivity (more characteristic of secondary psychopathy), and this psychoticism-impulsivity link is stronger in individuals with elevated BIS-FFFS scores (Heym and Lawrence, 2010). In the present study, the use of EPQ-RS enabled us to test whether psychoticism is negatively related to CI learning, as might be expected based on the fact that, using the same task variant, CI was found to be abolished in offenders with PDs (He et al., 2011).

Further predictions follow from Eysenck’s (1957, 1967) theory: for example, it suggests that the tendency for introverts to condition more readily than extraverts should be exacerbated by high neuroticism. This theory has been modified to take the nature of the US into account (Gray, 1970, 1972). For positive stimuli (as used in the present study), Eysenck’s theory predicts that conditioning will be better in those with higher levels of introversion, whereas Gray’s (1970) theory predicts that conditioning will be better in those with higher levels of extraversion. These predictions have been tested many times, but not in relation to CI.

In a previous study using a different inhibitory learning procedure, participants with higher BAS scores (specifically reward responsiveness, but not the other sub-scales) unexpectedly showed more rather than less CI (Migo et al., 2006). From a theoretical perspective, this is surprising in that higher BAS activity is predicted to increase conditioning to reward-related stimuli, and higher BIS activity conditioning to signals of non-reward (Corr et al., 1995; Pickering, 1997) – such as the absence of the expected rewarding outcome learned about in the CI task. Therefore we would predict that CI should have increased with BIS scores in this task – yet no such relationship was found (Migo et al., 2006). The present study used a larger sample to further explore the direction of the relationship between CI and those aspects of impulsivity measured by the BAS scales, and to reevaluate the prediction that increased BIS scores should be associated with higher levels of CI.

## Materials and Methods

### Design

The overall design of the experiment was identical to that used in previous studies (He et al., 2011, 2012), and employed Lego blocks as neutral CSs and positive and neutral International Affective Picture System (IAPS) pictures as reinforcement and non-reinforcement respectively. There were three stages: (1) pre-test; (2) training with elemental and compound stimuli; and (3) the test stage (Table 2). In the pre-test stage, participants were required to rate the stimuli and stimulus compounds to be used in the training and test stages, to establish whether differences in responding to the stimuli at test could be due to biases present before the start of training.

In the elemental training stage two CSs, A and C, were paired with reinforcement (A+ and C+ trials), while a further two, U and V, were paired with non-reinforcement. This training provided a measure of participants’ simple associative learning. It also established A and C as excitatory CSs signaling a positive outcome, which facilitated the subsequent establishment and detection of CI. An *a priori* exclusion criterion was applied based on elemental training performance: participants who failed to learn the simple discrimination between C+ and V− trials [i.e., rating scores (C−V) = <0^1^] were excluded from all subsequent analyses (with the exception of the correlational analyses performed to examine the relationships between the level of excitatory or of inhibitory learning and the age of the participants).

During the compound training stage, the AZ compound signaled reinforcement (AZ+), whereas AP signaled non-reinforcement (AP−). As A had been paired with reinforcement in the previous stage, presenting AP allowed P to signal the absence of the reinforcement otherwise indicated by A, and was thus expected to establish P as a conditioned inhibitor. Two additional stimulus compounds, CY and BX, were reinforced and non-reinforced respectively.

Although successful discrimination between AZ and AP would be consistent with the proposal that P was a conditioned inhibitor, it is not sufficient. For example, participants might respond more to AZ simply because Z was reinforced on every trial. In order to establish unequivocally that P was a conditioned inhibitor we conducted a summation test – more specifically, we examined whether P would suppress responding to a different excitatory stimulus more than would a suitable control stimulus (cf. Rescorla, 1969). The continued excitatory training with C on CY+ trials (C had also been reinforced in the previous stage) means it provided an excitatory test stimulus against which the inhibitory effects of P could be evaluated. The BX− trials were designed to establish X as a control stimulus which was presented the same number of times as P, and in a similar manner (in compound with another stimulus, and paired with non-reinforcement). However, the stimulus with which X was presented was novel so that X, unlike P, did not signal the absence of reinforcement during this training stage. Therefore X should not have acquired any inhibitory properties.

The test stage, like the pre-test, compared ratings of the stimuli and stimulus compounds that had signaled reinforcement (A, C, AZ, CY) and non-reinforcement (AP, BX), and also the test compounds (CP, CX). The critical comparison was between the test compounds CP and CX. Stimulus C was excitatory, and was predicted to elicit high ratings indicating expectation of reinforcement. If P was a conditioned inhibitor it should reduce this high rating to C, whereas the critical comparison stimulus, X, should not. CI would therefore be evident as lower ratings to CP than to CX. The identities of the stimuli used as P and X were counterbalanced across the participants, as were those of A and B (and C and V, see above).

### Participants

A total of 194 healthy participants took part in the computer-based learning task, all of whom completed the EPQ-RS and BIS/BAS questionnaires. The participants were recruited from the University of Nottingham (UK campus) and the local community. The participants included 98 males and 96 females, and the mean age of participants was 24.85, range 18–56. Eighteen out of 194 participants failed the excitatory associative learning task during the elemental training stage [i.e., rating scores (C–V) = <0 – see below], which was used as an exclusion criterion. The study was approved by the University of Nottingham, School of Psychology Ethics Committee. Participants received an inconvenience allowance of £3 cash to cover their travel expenses.

### Stimuli

**Figure 1:**
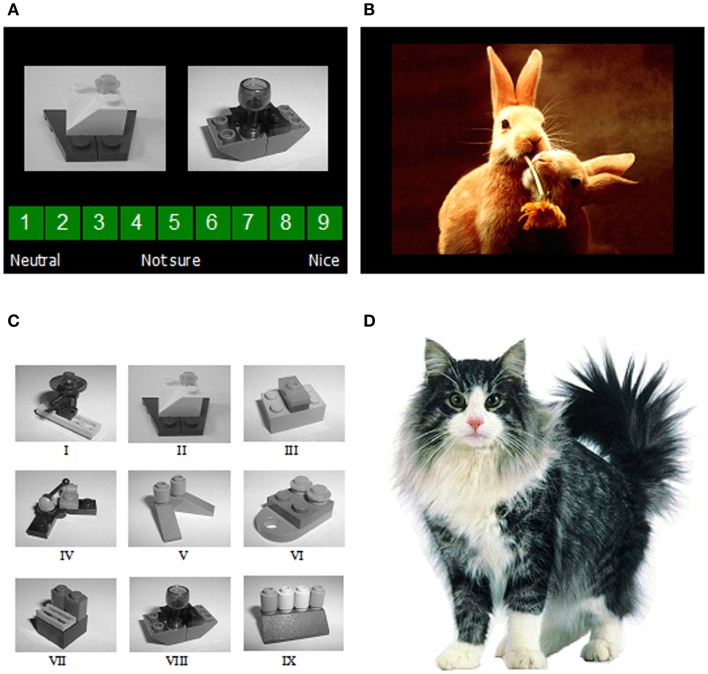
**(A)** Two examples of the image presentations used as conditioned stimuli, shown together with the rating scale used to guess or predict what valence of unconditioned stimulus (a positive or neutral IAPS image) Mogwai would bring; **(B)** an example of one of the image presentations used as the unconditioned stimuli; **(C)** the nine images of Lego blocks used as conditioned stimuli; **(D)** Mogwai the cat as presented prior to the unconditioned stimuli in the training stages.

Lego block pictures (*n* = 9) were used as the CSs (Figure 1). The USs were selected by a pilot study from the IAPS (Lang et al., 2005). The IAPS provides a set of images, standardized on the basis of participants’ ratings, on the dimensions of valence and arousal from 1 to 9, 1 representing a low rating on each dimension and 9 a high rating (i.e., 1 as low pleasure, low arousal). The USs in the present study included 10 positive pictures and 10 neutral pictures, excluding erotic pictures (see Table 1 for mean valence and arousal ratings of the images in use). Conditioning was measured using a rating scale: participants were asked to guess or predict what kind of picture would follow presentation of the Lego blocks using a rating scale from 1 (neutral) to 9 (positive), with the rating 5 to reflect uncertainty as to what kind of image was expected to follow.

**Table 1:** **The valence and arousal ratings of the IAPS images used**.

Images	Mean valence (SD; range)	Mean arousal (SD; range)
10 Neutral	4.94 (0.08; 4.86–5.08)	2.79 (0.54; 1.72–3.46)
10 Positive	7.80 (0.27; 7.49–8.28)	4.93 (1.07; 3.08–6.73)

### Questionnaires

The following were administered to the participants after the CI learning computer task.

#### Eysenck personality questionnaire revised short scale

The EPQ–RS is a 48 item yes/no questionnaire, suitable for the age range 16–70 years (Eysenck et al., 1985). It is used to assess dimensions of personality in relation to four factors: extraversion (E), psychoticism (P), neuroticism (N), and the response distortion (Lie) scale. There are 12 items for each factor.

#### Behavioral inhibition system/behavioral activation system scale

This consists of a list of 20 items for which participants use a four-point response scale to express whether the statement is true or false for them (Carver and White, 1994). The questionnaire divides in five sub-scales: BIS-anxiety, BIS-FFFS, BAS-drive, BAS-fun seeking, and BAS-reward responsiveness.

### Procedure

This was the same as that used previously (He et al., 2011, 2012) with some minor variations (reported in full below). Participants were invited to take part in a research study on learning using a computer-based task. Before the task, each participant had to read the information sheet and sign a consent form. The task instructions were that a cat “Mogwai” would bring participants either a positive picture or a neutral, boring picture, depending on what kind of Lego blocks she found in her basket (Figure 1). Participants were asked to guess or predict what kind of picture would follow presentation of the Lego blocks using the rating scale described above. Reminder instructions were presented on-screen at each stage of the procedure.

Before the start of the pre-test phase, participants were shown some example CSs and USs and further explanation was given as necessary. The samples of CS and US images were individually color printed on a 4.5 cm × 6 cm card and these pictures were representative of, but not subsequently used as, stimuli during the experiment. Participants were told that the whole computer-based experimental session would last about 20 min and comprise three stages. At the same time, they were shown an example of CS presentations with the rating scale, and were told that during the experiment they would need to click the corresponding number to guess or predict the valence of the US (a positive or a neutral picture) according to the different Lego blocks that had been presented. Participants were encouraged to ask questions at this stage. The three stages of the computer-based experimental session then followed.

#### Pre-test stage

During the first (pre-test) stage of the experiment, participants were told they must guess what kind of picture the cat might bring based on the Lego blocks presented, although the instructions specified that no pictures would follow. A Lego block CS was presented with the rating scale, until the participants clicked on a number button to guess the US valence; this triggered the next CS presentation, which followed immediately. In this and all subsequent stages of the experiment CS presentations were counterbalanced for right/left position on the screen across participants, and the various trial types were presented in a semi-random sequence (i.e., constrained only by the total number of trials of a particular type scheduled in each stage). In this stage there was a total of 16 presentations, two of each stimulus or stimulus combination presented (these being A, C, AZ, AP, BX, CY, CP, and CX; see Table 2).

**Table 2:** **The design of the experiment used in the third variant of the task**.

Pre-test		Elemental training		Compound training		Test	
CSs	No. of trials	CSs	No. of trials^1^	CSs ± outcome	No. of trials	CSs	No. of trials
**PHASE**							
A	2	A+	12	AZ+	8	A	2
C	2	U−	12	AP−	12	C	2
AZ	2	V−	12	BX−	12	AZ	2
AP	2	C+	12	CY+	8	AP	2
BX	2					BX	2
CY	2					CY	2
CP	2					CP	4
CX	2					CX	4

*In the pre-test all participants gave baseline ratings of the various stimuli. Letters denote the nine CSs (pictures of Lego blocks) which were counterbalanced (see text). “+” Denotes reinforcement (a positive IAPS picture) and “−” non-reinforcement (a neutral IAPS picture). ^1^Sixty two participants were tested with 8 rather than 12 elemental training trials. Compound training established P as a signal for the *absence* of reinforcement, rendering it inhibitory. In addition CY was reinforced, and BX non-reinforced. Thus C served as an excitatory cue against which the effect of the inhibitory P could be examined, while X served as a control for P. At test CP and CX were presented: to the extent that P was inhibitory, it would successfully counteract the tendency of C to predict reinforcement, relative to X*.

#### Training stages

On completion of the pre-test, the conditioning trials commenced and US presentations were introduced. The instructions were as before, but with the exception that participants were advised that following their guess they would be shown the picture that the cat had brought. The first training stage used the CS elements, and comprised six training blocks, each with two of each of the four kinds of trial (A+, U−, V−, and C+). As in the pre-test, the Lego block was presented until the participant clicked a number button to predict the valence of the US to follow, at which point a US, randomly selected from the pool of positive or neutral USs as appropriate, was shown on the screen for 1 s. This was followed by a 1 s gap, during which a picture of the cat Mogwai (around 6 cm × 6 cm) was presented in the middle of the screen on a white background. This sequence of events comprised a trial. The second, compound training stage followed directly after this training with the CS elements, and comprised four kinds of trial (AZ+, AP−, BX−, and CY+). There was a total of eight excitatory trials of each type in this stage; the number of inhibitory trials depended on the task variant (see below). The different trial types were analyzed in four equivalent blocks of trials.

#### Test stage

The test stage was exactly the same as the pre-test stage, except that there were four rather than two presentations of each of the critical test compounds CP and CX. As in the earlier stages of the experiment, there were on-screen reminders of the task instructions. Throughout the experiment, whenever participants asked questions or made comments they were asked to try to focus on the task and to try to remember or guess which outcome (positive or neutral picture) was predicted by the Lego blocks.

### Procedural variants

There were three variants on the experimental procedure used to test CI in the present study. In the first (*n* = 43) the pictures of the CSs were colored and the number of presentations of the non-reinforced compounds was eight (rather than 12 as shown in Table 2). The second refinement was identical to the first (*n* = 19), except that the colored CS images were changed to black and white pictures. The final variant (*n* = 132) differed only in that the number of non-reinforced compound presentations was increased from 8 to 12 (as in Table 2). This final version was that used in our previously published reports (He et al., 2011, 2012). These three procedural variants did not result in equivalent levels of CI, the third being the most effective. However, variation in the level of CI does not preclude investigation of its relationship to individual differences variables and – as would be expected – CI was clearly demonstrated over the sample as a whole.

### Analysis

The dependent variable was the mean rating given for each particular trial type, which was assessed in each training block of each stage. Statistical analyses of overall learning were by analysis of variance (ANOVA), with discrimination (e.g., A+ vs. U− and C+ vs. V−), reinforcement (reinforced or not), and trial block as within-subjects factors. Additionally, a summary measure of excitatory learning was provided by the difference in mean ratings on C and V trials during the initial training stage, i.e., C–V. As C was the excitatory stimulus, the greater the C–V score, the higher the level of excitatory learning. A summary measure of CI was provided by the difference between the mean ratings on CX and CP trials given during the test stage, i.e., CX–CP. P was the putative inhibitor, and thus supposed to suppress evaluation of C more than X; thus the higher the CX–CP score, the greater the inhibitory learning. Significant two-way interactions were explored with simple main effects analysis. Comparison of the summary learning scores in males vs. females was by *t*-test.

Correlational analyses were used to compare overall learning and questionnaire scores for EPQ and BIS/BAS sub-scales. Bonferroni adjustments can be employed to reduce the possibility of Type I errors when examining multiple correlation coefficients (Larzelere and Mulaik, 1977; Holm, 1979; Rice, 1989). However, particularly for statistically small effects, the likelihood of Type II error is increased (Perneger, 1998; Jennions and Møller, 2003; Nakagawa, 2004). Thus, unless otherwise stated, the correlations reported in this paper are corrected using Benjamini and Hochberg’s (1995) procedure, rather than Bonferroni which has less statistical power (so the uncorrected p values are reported in Table 3).

**Table 3:** **Correlations between the EPQ-RS and BIS/BAS variables, the demographic variables, and the measures of excitatory and inhibitory associative learning**.

	C–V	CX–CP	Age	Sex	L	P	E	N	FFFS	BIS	BAS-D	BAS-FS	BAS-RR
C–V		−0.01	0.22**	−0.10	0.03	0.07	0.07	−0.17*	0.01	−0.07	−0.21**	−0.11	−0.08
CX–CP	−0.14, 0.12		0.11	−0.18*	0.17	0.00	−0.03	−0.19*	−0.17*	−0.19*	0.08	0.03	−0.13
Age	0.08, 0.35	−0.02, 0.24		−0.19*	0.13	−0.03	−0.16	−0.36**	−0.10	−0.23**	−0.22**	−0.29**	−0.37**
Sex	−0.23, 0.03	−0.32, −0.04	−0.32, −0.04		0.18*	−0.16	0.19*	0.28**	0.32**	0.41**	0.05	0.00	0.10
L	−0.10, 0.16	−0.01, 0.24	−0.01, 0.26	0.04, 0.31		−0.08	0.04	−0.22**	−0.14	−0.11	−0.17	−0.16	−0.18*
P	−0.07, 0.19	−0.13, 0.13	−0.16, 0.10	−0.28, −0.03	−0.20, 0.05		0.04	−0.02	−0.12	−0.22**	0.08	0.27**	0.09
E	−0.07, 0.20	−0.16, 0.10	−0.28, −0.02	0.06, 0.32	−0.09, 0.16	−0.08, 0.16		−0.31**	−0.22**	−0.07	0.30**	0.43**	0.22**
N	−0.31, −0.03	−0.32, −0.05	−0.45, −0.19	0.14, 0.40	−0.34, −0.08	−0.14, 0.10	−0.43, −0.18		0.47**	0.54**	−0.04	−0.04	0.13
FFFS	−0.13, 0.14	−0.31, −0.03	−0.23, 0.04	0.19, 0.44	−0.26, −0.01	−0.24, 0.00	−0.35, −0.09	0.36, 0.57		0.53**	−0.18*	−0.12	0.08
BIS	−0.20, 0.06	−0.32, −0.05	−0.36, −0.09	0.29, 0.52	−0.23, 0.01	−0.34, −0.09	−0.19, 0.06	0.44, 0.63	0.42, 0.62		0.00	0.00	0.24**
BAS-D	−0.35, −0.07	−0.05, 0.20	−0.35, −0.07	−0.07, 0.18	−0.29, −0.04	−0.04, 0.20	0.16, 0.42	−0.16, 0.09	−0.31, −0.04	−0.14, 0.13		0.51**	0.53**
BAS-FS	−0.24, 0.02	−0.10, 0.16	−0.42, −0.15	−0.12, 0.13	−0.28, −0.04	0.14, 0.39	0.30, 0.53	−0.16, 0.08	−0.24, 0.01	−0.09, 0.16	0.40, 0.61		0.49**
BAS-RR	−0.21, 0.05	−0.25, 0.00	−0.49, −0.23	−0.03, 0.22	−0.30, −0.04	−0.04, 0.21	0.09, 0.32	0.00, 0.25	−0.05, 0.21	0.10, 0.37	0.42, 0.63	0.37, 0.59	

*N* = 176. *p < 0.05, **p < 0.01 (uncorrected). Above the diagonal the correlation coefficient, *r*. Below the diagonal, the lower and upper limits of the 95% confidence interval of the correlation coefficient. C–V, excitatory associative learning; CX–CP, inhibitory associative learning; L, Lie (response distortion); P, psychoticism; E, extraversion; N, neuroticism; FFFS, BIS fight, flight, freeze sub-scale; BIS, behavioral inhibition system anxiety sub-scale; BAS-D, BAS-drive sub-scale; BAS-FS, BAS-fun seeking; BAS-RR, BAS-reward responsiveness.

## Results

### Conditioned inhibition confirmed by summation test

#### Pre-test stage

There was little difference on the rating scores of the stimuli prior to conditioning (all being around five). Importantly, there was no significant difference in responding to the two critical test compounds (CP vs. CX), *F* < 1.

#### Pre-training stage and training stage

**Figure 2:**
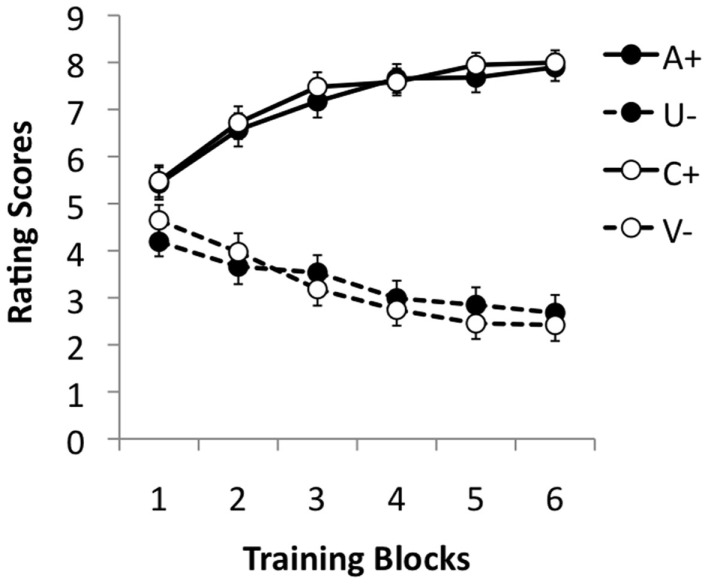
**Mean rating scores for A+, U−, V−, and C+ during the six training blocks of the pre-training stage**. A rating of 9 reflects expectation of a positive image, 1 of a neutral image, and 5 uncertainty; 95% confidence intervals are presented.

During the pre-training stage, the ratings of A and C steadily increased, while those to the U and V stimuli fell gradually, suggesting that the participants learned both discriminations in this phase (see Figure 2). This impression was supported by statistical analysis. ANOVA with discrimination (A/U vs. C/V), reinforcement and pre-training block (1–6) as factors revealed a significant three–way interaction, *F*(5, 875) = 2.70, *p* = 0.02, $\eta_{p}^{2}=0.015$. The main effects of block and reinforcement were significant, *F*(5, 875) = 4.80, *p* < 0.001, $\eta_{p}^{2}=0.027$, and *F*(5, 175) = 465.68, *p* < 0.001, $\eta_{p}^{2}=0.727$, respectively. Moreover, these two factors interacted significantly, *F*(5, 875) = 119.07, *p* < 0.001, $\eta_{p}^{2}=0.405$. The effect of discrimination was not significant, *F* < 1, nor the interaction between block and discrimination, *F*(5, 875) = 1.77, *p* = 0.12, $\eta_{p}^{2}=0.01$. The interaction between discrimination and reinforcement was not significant, *F*(1, 175) = 1.57, *p* = 0.211, $\eta_{p}^{2}=0.009$.

To explore the three-way interaction further ANOVAs were performed separately on the two discriminations. These revealed a significant interaction between reinforcement and discrimination for both the A/U and C/V discriminations, *F*(5, 875) = 355.05, *p* < 0.001, $\eta_{p}^{2}=0.239$, and *F*(5, 875) = 83.51, *p* < 0.001, $\eta_{p}^{2}=0.323$, respectively. Simple main effects analysis revealed that the effect of reinforcement was highly significant on all training blocks in both discriminations, smallest *F*(1, 175) = 12.36, *p* = 0.001, $\eta_{p}^{2}=0.066$, for block 1 of the C/V discrimination. The main effect of block was also significant for both reinforced and non-reinforced trials in both discriminations, smallest *F*(5, 875) = 16.07, *p* < 0.001, $\eta_{p}^{2}=0.084$, for U trials.

**Figure 3:**
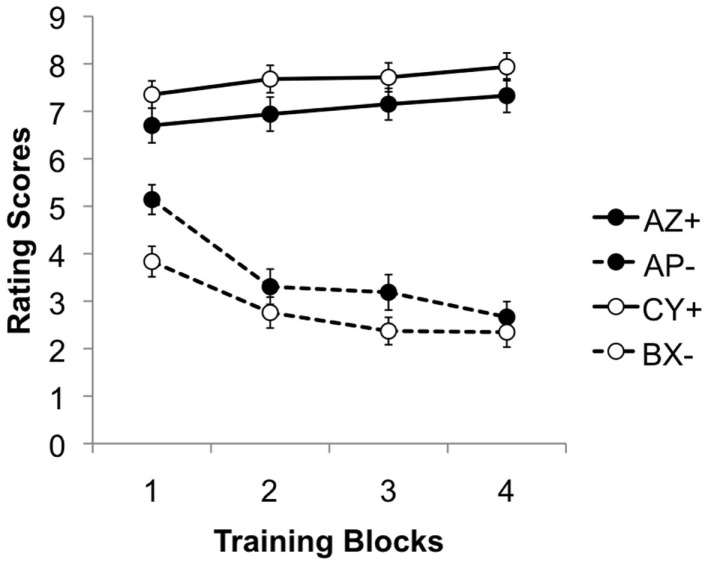
**Mean rating scores for AZ+, AP−, BX−, and CY+ during the four blocks of the training stage**. A rating of 9 reflects expectation of a positive image, 1 of a neutral image, and 5 uncertainty; 95% confidence intervals are presented.

During the training stage, the ratings of AZ and CY steadily increased, while those of AP and BX fell gradually (see Figure 3), again suggesting that both discriminations were learned successfully. This impression was again confirmed by statistical analysis. An ANOVA with discrimination (AZ/AP vs. CY/BX), reinforcement and training block (1–4) as factors, revealed a significant three–way interaction, *F*(3, 525) = 74.54, *p* < 0.001, $\eta_{p}^{2}=0.299$. The main effects of block and reinforcement were significant, *F*(3, 525) = 29.80, *p* < 0.001, $\eta_{p}^{2}=0.146$, and *F*(1, 175) = 45.58, *p* < 0.001, $\eta_{p}^{2}=0.214$, respectively. Moreover, these two factors interacted significantly, *F*(3, 525) = 3.15, *p* = 0.025, $\eta_{p}^{2}=0.018$. The effect of discrimination was not significant, *F* < 1, but the interactions between discrimination and both block and reinforcement were significant, *F*(3, 525) = 3.53, *p* = 0.015, $\eta_{p}^{2}=0.02$, and *F*(1, 175) = 480.34, *p* < 0.001, $\eta_{p}^{2}=0.733$ respectively.

Further ANOVAs were conducted to explore the three-way interaction further. These confirmed a significant interaction between block and reinforcement for both discriminations, smallest *F*(3, 525) = 33.95, *p* < 0.001, $\eta_{p}^{2}=0.162$, for the CY/BX discrimination. Simple main effects analysis revealed that the effect of reinforcement was significant for both discriminations on every block, smallest *F*(1, 175) = 39.57, *p* < 0.001, $\eta_{p}^{2}=0.184$, for the first block of the AZ/AP discrimination. In addition the effect of blocks was significant for both reinforced and non-reinforced trials in both discriminations, smallest *F*(3, 525) = 3.96, *p* = 0.008, $\eta_{p}^{2}=0.022$ for AP trials.

#### Test stage

**Figure 4:**
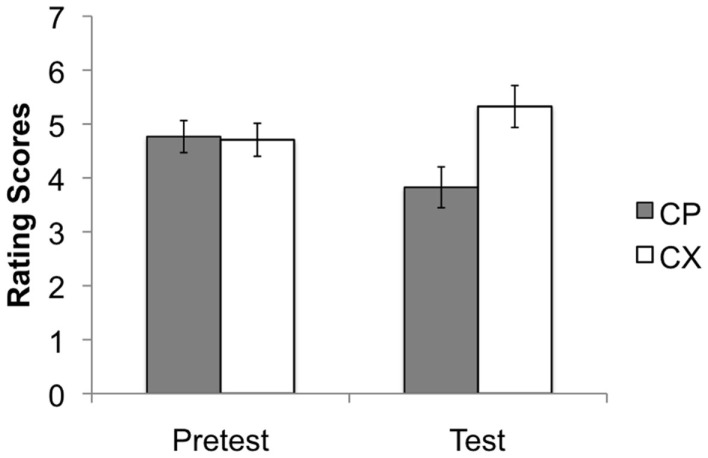
**Mean rating scores for the key comparison stimulus compounds CP and CX during the pre-test and test stages**. A rating of 9 reflects expectation of a positive image, 1 of a neutral image, and 5 uncertainty; 95% confidence intervals are presented. The stimulus compounds elicited similar ratings prior to conditioning, but the test ratings confirmed the presence of conditioned inhibition, evident as lower ratings to CP than to CX.

Figure 4 shows the rating scores during the test stage. Here the critical comparison was between ratings of CP and CX during the pre-test and the test stages. It can be seen from Figure 4 that the rating of CP was noticeably lower than CX during the test. This difference was confirmed by statistical analysis: an ANOVA with stage (pre-test and test), and stimulus (CP vs. CX) as factors revealed no effect of stage, *F* < 1, but a significant effect of stimulus, *F*(1, 175) = 22.95, *p* < 0.001, $\eta_{p}^{2}=0.116$. There was also a significant interaction between these two factors, *F*(1, 175) = 22.65, *p* < 0.001, $\eta_{p}^{2}=0.115$. Simple main effects confirmed that participants gave significantly lower rating scores to CP than to CX during the test stage, *F*(1, 175) = 49.79, *p* < 0.001, $\eta_{p}^{2}=0.183$ but not at the pre-test stage, *F* < 1. The results confirm the overall conclusion that P had become a conditioned inhibitor.

### Demographic characteristics and learning differences

In general, males performed better than females, as reflected in the summary measures of both excitatory, *t*(192) = 2.08, *p* = 0.04, and inhibitory learning, *t*(174) = 2.44, *p* = 0.02. There was also a significant correlation between the age of the participants and the summary measure of excitatory learning (C–V), *r*(194) = 0.18, *p* = 0.01. However, there was no correlation between age and the summary measure of inhibitory learning, *r*(174) = 0.11, *p* = 0.14.

### The relationship between excitatory and inhibitory learning

The correlation between the rating scores for (C–V) and (CX–CP) was examined directly. The results showed that there was no significant correlation between the two ratings, *r*(194) = 0.12, *p* = 0.09, suggesting that – despite their inevitable interdependence – individual differences in inhibitory learning are not entirely dependent on differences in excitatory learning.

### Individual differences in excitatory learning

#### Eysenck personality questionnaire revised short scale

There was a significant negative correlation between the EPQ-RS neuroticism scores and the summary measure of excitatory learning (C–V), *r* = −0.17, *p* = 0.021 (see Table 3). However, the correlations between excitatory learning and psychoticism and extraversion were not significant.

#### Behavioral inhibition system/behavioral activation system scale

There was a significant negative correlation between the BAS-drive scores and the summary measure of excitatory learning (C–V), *r* = −0.21, *p* = 0.004. However, there were no further significant correlations between the other sub-scales of the BIS/BAS and excitatory learning (C–V, see Table 3).

### Individual differences in inhibitory learning

#### Eysenck personality questionnaire revised short scale

There was a significant negative correlation between the EPQ-RS neuroticism scores and the summary measure of inhibitory learning (CX–CP), *r* = −0.19, *p* = 0.013. However, there were no significant correlations between the other sub-scales of the EPQ-RS and CX–CP (see Table 3).

#### Behavioral inhibition system/behavioral activation system scale

There were significant negative correlations between the BIS-anxiety scores (*r* = −0.19, *p* = 0.013) and BIS-FFFS (*r* = −0.17, *p* = 0.021) scores and the summary measure of inhibitory learning (CX–CP). However, there were no significant correlations for the BAS sub-scales and CX–CP (see Table 3).

### Demographic and individual differences variables joint effects on excitatory and inhibitory learning

To take into account the observation that both age and sex are related to the individual difference variables as well as the learning measures two multiple linear regressions were conducted using the inhibitory and excitatory learning measures as the criterion variables. The predictor variables were the demographic variables and the individual difference variables associated with the EPQ-RS and BIS/BAS measures.

Taken together the multiple-R for the measure of excitatory learning was 0.37 (*R*^2^ = 0.13) which was significant (*p* = 0.007). However, only BAS-drive had a statistically significant unique relationship with the excitatory learning measure (β = −0.24, $r_{p}^{2}=0.04$, *p* = 0.01), accounting for less than one third of the variability that the overall equation accounts for. The reason for neuroticism not showing a unique relationship is likely to be because of its relatively high correlations with both BIS-revised and FFFS as well as age and sex of the participants (see Table 3). For the measure of inhibitory learning the multiple-R was 0.31 (*R*^2^ = 0.10). This was not statistically significant (*p* = 0.07). Similarly, none of the demographic, EPQ-RS or BIS/BAS variables was individually statistically significant. This suggests that while the zero order correlations demonstrate relationships between some of the demographic and individual difference variables and the inhibitory learning measures the covariance of subsets of the predictor variables is sufficiently high to be partialed out as part of the linear regression procedure, leading to an underestimation of the relationship between individual predictor variables and the criterion variable.

## Discussion

As might be expected, using an established procedure (He et al., 2011, 2012) CI was robustly demonstrated in this large sample of participants in a summation test. What the present study adds to this prior work is clarification of how individual variations in inhibitory and excitatory learning relate to established individual difference measures. Specifically we examined participants’ neuroticism, extraversion, and psychoticism, as well as behavioral inhibition and behavioral activation, as proposed by the personality theories of Eysenck (1957, 1967, 1981), Eysenck et al. (1985), Gray (1972, 1982), and Gray and McNaughton (2000). These biologically based personality theories should most closely relate to associative learning theories derived from the study of animal behavior.

We found that those with higher EPQ-RS neuroticism showed reduced levels of both excitatory and inhibitory conditioning (as reflected in the C–V and CX–CP scores respectively). Reduced excitatory learning was also found in those with higher BAS-drive, but here there was a dissociation, in that inhibitory learning was not affected by this measure but was instead negatively related to both BIS-FFFS and BIS-anxiety.

Thus, as might be expected given the dependence between excitatory and inhibitory learning, both were attenuated in those with higher neuroticism. Similarly, as might be expected given the relationship between neuroticism and BIS, inhibitory learning was also related to the BIS scores. The correlations found here between the EPQ-RS and the BIS/BAS sub-scales largely replicate those earlier reported (Table 3; Heym et al., 2008). Thus the findings are consistent with higher levels of neuroticism being accompanied by generally impaired associative learning. There was also evidence for some dissociation in the effects of behavioral activation and behavioral inhibition on excitatory and inhibitory learning respectively.

However, contrary to what might seem to follow from the original version of Gray’s (1972, 1982) theory, we found that higher scores on the BIS scale were correlated with *impaired* rather than facilitated inhibitory learning. Clinical observations are consistent with elevated behavioral inhibition in anxiety disorders (Barlow, 2000), and according to Gray (1972, 1982) the BIS is activated by signals of punishment, signals of non-reward, and innate fear stimuli. It should be noted that Gray’s behavioral inhibition theory is not a theory of Pavlovian CI as such. However, there is overlap in the sense that signals of non-reward should excite the BIS (whereas signals of non-punishment excite the behavioral activation system and result in an emotional state more akin to relief). Since the present task was appetitively motivated (using positive IAPS images), the conditioned inhibitor is equivalent to a signal of non-reward and would be expected to engage the BIS.

Thus in a general sense, the present results suggest that habitual overactivity in the BIS in those high in the related temperamental trait can impair its normal function. According to the revised version of the theory (Gray and McNaughton, 2000; Corr, 2010) BIS-anxiety mediates the detection and resolution of goal conflict (for example between approach and avoidance, by way of “risk assessment” behaviors) rather than reactions to conditioned aversive stimuli, which are mediated by the BIS-FFFS. Signals of non-reward are secondarily aversive, but are a less likely trigger for the BIS-FFFS than are signals of punishment, and are more likely to engage the BIS-anxiety system. In any event, in the present study both BIS-FFFS and BIS-anxiety were negatively related to inhibitory learning, so the general conclusion still stands: temperamentally high levels of BIS activation were associated with impaired rather than enhanced BIS functioning.

Another surprising finding was the lack of any correlation between measures of excitatory or inhibitory learning and extraversion, which is inconsistent with Eysenck’s (1957, 1967) theory of how differences in conditionability give rise to differences in personality. There are grounds to suppose that conditioning differences will also depend on the nature of the US for positive stimuli (as used in the present study), but this should just affect the direction of difference, with higher rather than lower conditioning predicted in extraverts (Gray, 1970, 1972).

The results of the present study are likely to be robust in that the sample size was relatively large. However, to draw stronger conclusions ideally the experiment should be replicated using a different task variant, to exclude the possibility that there could be some artifact in consequence of the use of a single procedure. In particular, the inhibitory learning procedure used in the present study uses positive IAPS images as the US. The negative images are both more salient and would be predicted to show a different pattern of interrelationships with BIS/BAS scores.

Finally, males generally performed better than females, as reflected in their higher overall scores for both excitatory and inhibitory learning. This sex difference is consistent with the finding that both excitatory and inhibitory learning are reduced in those with higher neuroticism scores – as it is very well-established that females show higher levels of neuroticism (Jorm, 1987; Francis, 1993; Lynn and Martin, 1997), as well as higher levels of BIS-anxiety (Gray, 1971). Both of these sex differences were confirmed in the correlational analyses reported in Table 3 (the correlations go in the predicted direction in that females are coded higher than males in the data file). Thus the females tested in the present sample were more neurotic and showed higher behavioural inhibition than did the males.

There was also a significant correlation between age and associative learning, in that older participants showed relatively better excitatory learning, although inhibitory conditioning did not vary with age (also it should be noted that this was a relatively young sample – in the range 18–56 years).

### Comparison with earlier studies

The overall pattern of results is consistent with a role for impulsivity, as measured by BAS-drive, in excitatory but not inhibitory learning, and for behavioral inhibition in inhibitory but not excitatory learning. A number of previous studies have demonstrated apparently opponent effects using measures of impulsivity and behavioral inhibition, e.g., using the Go/No-Go task and the Stop Signal task (Visser et al., 1996; Logan et al., 1997; Enticott et al., 2006). However, to date there has been little systematic examination of the relationship between impulsivity and associative learning. The present results are consistent with the possibility that impaired associative learning processes could be responsible for aspects of impulsive behavior and disorders (He et al., 2011, 2012).

However, contrary to our predictions, the present study did not find any correlation between impulsivity (as measured by the BAS) and inhibitory learning performance, although inhibitory learning was related to BIS scores. This contrasts with our previous findings using a different task variant (Migo et al., 2006), where we found a negative correlation between inhibitory learning and BAS-reward responsiveness, but none with behavioral inhibition as measured by BIS scores. There are several possible explanations of these discrepancies. First, the sample was much smaller in the earlier study (Migo et al., 2006, which used 60 participants), thus there was less statistical power. Moreover, not only are the correlations between paper-and-pencil questionnaire measures and behavioral measures of impulsivity relatively low (Paulsen and Johnson, 1980; Milich and Kramer, 1984; Helmers et al., 1995; Claes et al., 2006), but it has also been argued that the low arousal conditions typical of laboratory testing underestimate impulsivity (Helmers et al., 1997). There were also procedural differences: in the earlier variant, stimuli were presented serially and included distractors, to reduce the potential role of external inhibition as an alternative explanation of disrupted responding when the inhibitory stimulus was introduced (Migo et al., 2006). By contrast, the present design controlled for external inhibition explicitly with the non-reinforced control stimulus, X.

### Small effect sizes for personality

Although statistically some associations were demonstrated, the effect sizes were relatively small. Yet the experimental design used in the present study has been used to demonstrate CI deficits in disordered groups with much smaller sample sizes. Specifically CI was clearly impaired in a sample of 24 non-psychotic offenders with PDs (He et al., 2011). We also found CI to be significantly reduced in a sample of 25 community-based schizophrenic participants, although with a different profile to that seen in offenders in that excitatory learning was also reduced (He et al., 2012). The study of offenders included dimensional scores from the International PD Examination (Loranger et al., 1994) and the Psychopathy Check List-Revised (Hare, 1991). There was no significant correlation between any of the available measures of personality or behavioral traits and the summary measures of excitatory and inhibitory learning. However, some of the effect sizes for these non-significant correlations were moderate and – despite the relatively modest sample sizes – clear group differences in relation to dangerousness and severity were demonstrated (He et al., 2011). In the study of CI in relation to schizophrenia, individual differences in symptomatology were captured by the Positive and Negative Syndrome Scale (PANSS; Kay et al., 1987). We found a significant correlation between the negative symptoms sub-scales of this measure and the summary measure of inhibitory learning, and also a marginally significant correlation with the excitatory learning score. In both cases the effect size was medium-large – this despite the fact that PANSS scores were not available for all participants (He et al., 2012).

### Implications for disorder

The results of the present study can be related to earlier studies of anxiety-related disorders. For example, the significant negative correlation between excitatory learning performance and EPQ-RS neuroticism suggests that individuals who are prone to suffer strong, changeable mood, and to overreact in emotional situations, show poorer excitatory learning ability. People who score higher on neuroticism have been argued to be more likely to experience anxiety (Eysenck, 1957, 1967), particularly if their extraversion scores are also low (Gray, 1970, 1972). In this sense, the results of the current study are consistent with the impaired associative learning processes seen in anxiety and depressive disorders (Fowles, 1980, 1993; Gray, 1985; Davey, 1992; Grillon, 2002). The present study extends the demonstration of impaired associative learning processes to inhibitory conditioning, which was also reduced in those with higher EPQ-RS neuroticism and higher BIS scores. Thus, the results point to (susceptibility to) anxiety as a predictor of impaired CI.

To date, we have been unable to recruit participants with clinical levels of anxiety disorder in sufficient numbers. However, the apparent relationship to anxiety demonstrated in the present study of normal participants is consistent with our finding of reduced inhibitory and excitatory learning in participants with schizophrenia (He et al., 2012). Patients with schizophrenia have been found to have relatively high BIS scores. Moreover, this questionnaire study showed that higher BIS sensitivity correlated with duration of illness (Scholten et al., 2006). However, we have no basis to comment on anxiety levels in the group of offenders we studied using this same task (He et al., 2011), and in the present study there was no relationship between inhibitory learning scores and psychoticism (which has been argued to predict psychopathic tendencies, Eysenck and Eysenck, 1976b; Eysenck, 1992).

### The relationship between excitatory and inhibitory learning

Inhibitory and excitatory learning are inevitably inter-dependent, since a conditioned inhibitor signals the absence of an outcome predicted by an excitatory stimulus. Thus excitatory learning must first be established before inhibitory learning is introduced. Indeed in the present study, in total 18 participants were excluded from the CI test because they did not meet the excitatory learning criterion. Given this background, some commonalities in the individual differences profile predicting better excitatory and those predicting better inhibitory learning is to be expected.

However, animal studies nonetheless suggest that inhibitory and excitatory learning are dissociable (Rescorla, 1969; Daw et al., 2002), and that positive and negative prediction error are coded opponently at the neuronal level (Tobler et al., 2003). Thus distinct neural substrates could underlie the variation in excitatory and inhibitory learning accompanying differences in neuroticism and behavioral inhibition in the present study (see also He et al., 2011, 2012). Moreover, the overall correlation between excitatory and inhibitory learning scores was not significant in the present study, suggesting that – despite their inevitable dependence on earlier excitatory conditioning – individual differences in inhibitory learning are not entirely dependent on those seen in excitatory learning.

### Implications for general theories of associative learning

Variations in excitatory and inhibitory learning could in principle be used to account for differences between people, but the available learning theories are monolithic. In other words, theories of associative learning are not yet sufficiently articulate to accommodate the effects of individual differences in information processing, in turn based in individual differences in nervous system function. The results reported in the present study underscore the importance of this kind of theoretical development, but the work needed is more complex than modeling a group difference in terms of an existing theory. Temperamental traits are measured as scores on continuous variables and the full complexity of an individual’s personality can only be captured as a profile of scores on a variety of measures, some of which are orthogonal, some of which are inter-dependent. Thus, for example, neuroticism and extraversion were originally conceived as orthogonal factors (Eysenck, 1957, 1967; 1981; Eysenck et al., 1985; Eysenck and Eysenck, 1991), as were behavioral inhibition and activation (Gray, 1972, 1982). However, since the latter reflect a rotation of Eysenck’s personality dimensions, neuroticism is correlated with behavioral inhibition and extraversion is correlated with behavioral activation (Gray, 1972, 1982). Similarly, as might be expected given that they are derived from a single scale, BIS-anxiety and BIS-FFFS are inter-dependent (Heym et al., 2008). Thus the formal inclusion of individual differences into contemporary theories of associative learning will require the introduction of multi-factorial moderating variables, to specify their effects on learning rate parameters such as the CS and US factors which influence associability.

Historically the aim has been to establish general laws of learning. The observed dissociation in the effects of behavioral activation and behavioral inhibition on excitatory vs. inhibitory learning could in principle be incorporated into learning theories which make formal predictions about inhibitory as well as excitatory learning (e.g., Rescorla and Wagner, 1972). This would not affect the generality of the theories and could improve their predictive power. However, the formal inclusion of reinforcement sensitivity theory (Gray, 1972, 1982; Gray and McNaughton, 2000) would suggest the need for different variants of the models to be applied to learning situations which use appetitive vs. aversive USs. Moreover, any such learning models would need to be weighted to take effect size into account, and effect sizes of the magnitude reported here could be too small to warrant what might be viewed as unnecessary complication. Ultimately, dynamic interactionist models would be necessary to capture the three-way interaction between personality, conditionability, and environmental context (Ferguson et al., 2012; Haslam et al., 2012).

## Conflict of Interest Statement

The authors declare that the research was conducted in the absence of any commercial or financial relationships that could be construed as a potential conflict of interest.

## Footnotes

^1^Only C and V were used for this purpose as the identities of Lego blocks serving as C and V were fully counterbalanced, whereas those of A and U were not.
